# Supplementary material for: Development of an H&E on-block staining technique for collagen detection in cryo-fluorescence tomography imaging of frozen breast tissue samples
Source: PLoS One. 2025 Jun 9;20(6):e0324493. doi: 10.1371/journal.pone.0324493 (PMC12148110; doi:10.1371/journal.pone.0324493)
Supplement: S1 File — (DOCX) [file pone.0324493.s001.docx]

**Breast Specimen Preparation**

1. Embed excised breast tissue in OCT (optimal cutting temperature).

1.1 (optional) Place squid ink spaghetti noodles in the mold with the OCT to act as fiducial markers.

1. Freeze at -20°overnight.

**CFT Imaging Protocol**

1. Set up Xerra (EMIT Imaging, Baltimore, MD) imaging study. We used a GFP filter and obtained images in 50 µm sections.
2. Reconstruct and register images using Vivoquant image processing software.

**H&E On-Block Staining Protocol**

1. At planned sectioning intervals, remove the OCT specimen block from the Xerra cryo-chamber.
2. Place a layer of medical gauze over the block, covering the tissue block face.
3. Use a disposable pipette to cover the entire block face with hematoxylin.
4. Allow the stain to penetrate the tissue for 60 sec.
5. Remove the gauze, tilt the block at an angle, and rinse the block face with deionized water, using a wash bottle.

10. Apply a new layer of medical gauze.

11. Use another pipette to cover the surface of the block with eosin.

12. Allow this stain to penetrate for 30 sec.

13. Rinse the block at an angle again, this time with 95% ethanol three times.

**Microscope Imaging**

14. Acquire images with the Omano OM2300S-V1 stereo-dissecting microscope and the Summit SK2-14X camera at the relevant magnifications. Use a ring light to illuminate the sample.

15. After imaging, refreeze the OCT sample (approx. 15 min. at -20°). We found that the block can sit for approximately 30 minutes before melting to a considerable degree where imaging is impacted.

**Gold Standard Histology Imaging**

16. Specimens that were not processed by Xerra should undergo typical H&E histological staining. The sample(s) should be embedded in OCT onto a chuck and sectioned with a standard cryostat. Our tissue was sectioned into 35 µm slices.

17. Slices should then be transferred to charged microscope slides.

18. Acquire images with the Leica Aperio AT2 digital slide scanner.

**Staining Technique Assessment**

19. An experienced pathologist should evaluate both the gold-standard and the on-block imaging data set for comparisons.
