# Supplementary material for: Development of an H&E on-block staining technique for collagen detection in cryo-fluorescence tomography imaging of frozen breast tissue samples
Source: PLoS One. 2025 Jun 9;20(6):e0324493. doi: 10.1371/journal.pone.0324493 (PMC12148110; doi:10.1371/journal.pone.0324493)
Supplement: S2 Fig — After staining the block-face, the sample was returned to the cryo-chamber, and sectioning continued. Each successive 50-µm slice was counted until all the stain was removed and a new block-face was revealed. Some samples underwent multiple staining trials. A one-sample t-test was performed to compute descriptive statistics. (A) is a visual representation of the data in the form of a XY plot. Created with GraphPad Prism. (PDF) [file pone.0324493.s002.pdf]

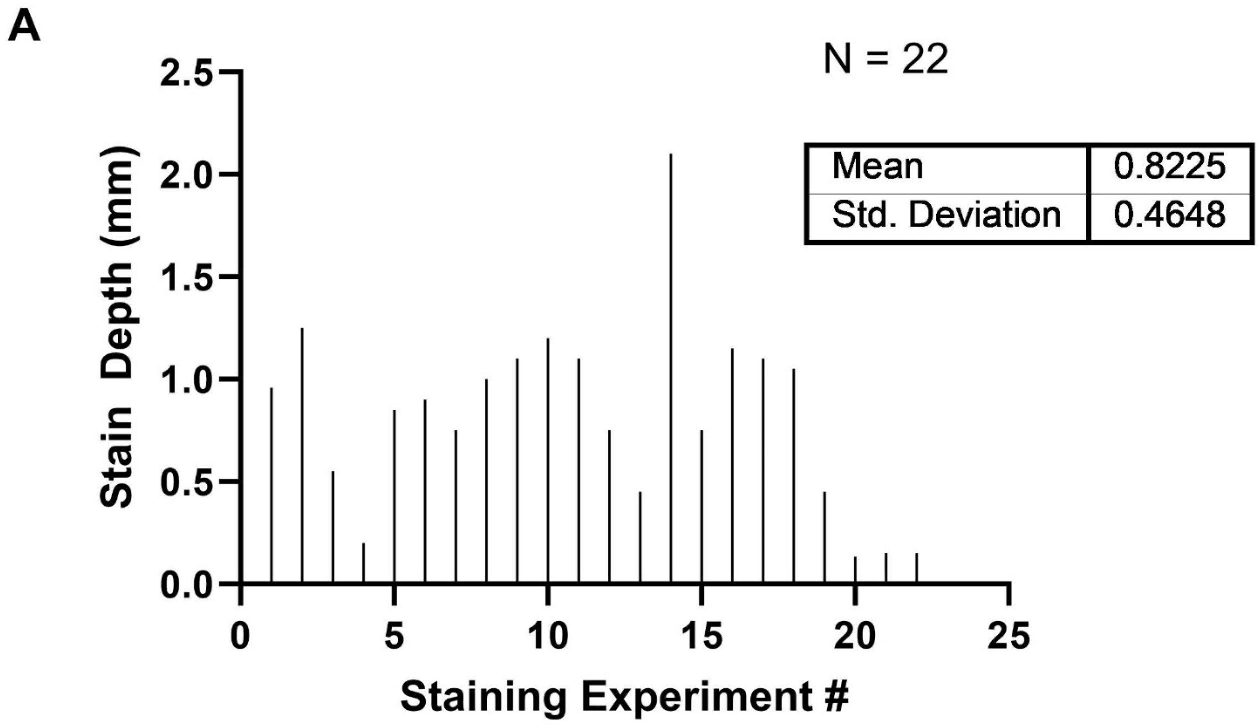

Figure S2: The reported stain penetration depths for 22 optimization experiments. After staining the block-face, the sample was returned to the cryo-chamber, and sectioning continued. Each successive 50-µm slice was counted until all the stain was removed and a new block-face was revealed. Some samples underwent multiple staining trials. A one-sample t-test was performed to compute descriptive statistics. **(A)** is a visual representation of the data in the form of a XY plot. Created with GraphPad Prism.
