# Supplementary material for: Development of an H&E on-block staining technique for collagen detection in cryo-fluorescence tomography imaging of frozen breast tissue samples
Source: PLoS One. 2025 Jun 9;20(6):e0324493. doi: 10.1371/journal.pone.0324493 (PMC12148110; doi:10.1371/journal.pone.0324493)
Supplement: S1 Table — We have incorporated a few suggestions to address a few common problems that one may encounter when trying to adapt this protocol for their own histology experiments. (PDF) [file pone.0324493.s003.pdf]

Tips and Troubleshooting

Issue:	Solution/Tip:
There is difficulty with staining larger, embedded samples.	Individuals staining larger specimen-embedded blocks may find it laborious to distribute the stains across the tissue face with a small pipette. To simplify the process, we have found that dipping a few sheets of gauze into the stain and lightly "stamping" it on the tissue face can be a quick and simple alternative.
The stain appears too light/dark.	For those who prefer a lighter/darker stain, we recommend adjusting the timing of stain penetration for hematoxylin and eosin in 30-second and 15-second increments, respectively.
The block needs to be stored overnight.	To ensure that the sample block does not dry out, it is best to store it in a resealable plastic bag inside a -20°C freezer overnight.
Staining results are inconsistent.	There are certain quality control measures that can be enacted to ensure consistent results. It is important to maintain fresh reagent quality to guarantee uniform staining. It can be helpful to filter the hematoxylin regularly to remove precipitates that can cause background staining. Additionally, standardized timing is the key to avoiding under- or over-staining. We recommend using a timer to help with this.

Table S1: Tips and Troubleshooting. We have incorporated a few suggestions to address a few common problems that one may encounter when trying to adapt this protocol for their own histology experiments.
